# Nearly Efficiency-Droop-Free AlGaN-Based Ultraviolet Light-Emitting Diodes with a Specifically Designed Superlattice p-Type Electron Blocking Layer for High Mg Doping Efficiency

**DOI:** 10.1186/s11671-018-2539-9

**Published:** 2018-04-24

**Authors:** Zi-Hui Zhang, Sung-Wen Huang Chen, Chunshuang Chu, Kangkai Tian, Mengqian Fang, Yonghui Zhang, Wengang Bi, Hao-Chung Kuo

**Affiliations:** 1 Institute of Micro-Nano Photoelectron and Electromagnetic Technology Innovation, School of Electronics and Information Engineering, Hebei University of Technology, Key Laboratory of Electronic Materials and Devices of Tianjin, 5340 Xiping Road, Beichen District, Tianjin, 300401 People’s Republic of China; 2 Department of Photonics and Institute of Electro-optical Engineering, National Chiao Tung University, Hsinchu, 30010 Taiwan

**Keywords:** DUV LED, Superlattice p-EBL, Hole injection, Electron leakage, Efficiency-droop-free

## Abstract

This work reports a nearly efficiency-droop-free AlGaN-based deep ultraviolet light-emitting diode (DUV LED) emitting in the peak wavelength of 270 nm. The DUV LED utilizes a specifically designed superlattice p-type electron blocking layer (p-EBL). The superlattice p-EBL enables a high hole concentration in the p-EBL which correspondingly increases the hole injection efficiency into the multiple quantum wells (MQWs). The enhanced hole concentration within the MQW region can more efficiently recombine with electrons in the way of favoring the radiative recombination, leading to a reduced electron leakage current level. As a result, the external quantum efficiency for the proposed DUV LED structure is increased by 100% and the nearly efficiency-droop-free DUV LED structure is obtained experimentally.

## Background

Ultraviolet beams in the wavelength regime of 200 nm~280 nm have found potential applications in water purification system [1, 2]. Considering the low DC driving voltage and the more compatibility with the water purification system, AlGaN-based deep ultraviolet light-emitting diodes (DUV LEDs) are selected as the excellent candidate. It is worthy of mentioning that treating the water with a big volume requires the purification system to provide the high-power UVC light source. However, the external quantum efficiency (EQE) for AlGaN-based DUV LEDs with the emission wavelength shorter than 280 nm is not satisfied at this moment [3]. The major limiting factor for the poor EQE partly arises from the large threading dislocation density (TDD) in the Al-rich quantum wells [2, 3]. The internal quantum efficiency (IQE) quickly decreases once the TDD is in the order of 10^9^ cm^−2^ [3]. Even if the TDD is reduced to the order of 10^8^ cm^−2^ that can enable the IQE of 60~80%, the effect of the efficiency droop may cause the EQE to be lower than 5% for the bare UVC LEDs when the injection current density exceeds 80 A/cm^2^ [4]. Note, the light extraction efficiency (LEE) for bare UVC LED chips is ~ 10% according to FDTD calculations [5]. One of the leading interpretations for the efficiency droop of III-nitride-based LEDs is the electron spillover into the p-type hole injection layer [6]. The Al-rich AlGaN p-type hole injection layer possesses a free hole concentration even lower than 1 × 10^17^ cm^−3^ [7], tending to cause a severer electron leakage level. Mehnke et al. have measured the parasitic emission that takes place in the p-type hole injection layer and the parasitic emission is well attributed to the electron leakage [8]. To reduce the electron escape from the multiple quantum wells (MQWs), one can increase the electron capture rate by inserting single spike layers in the quantum barriers [9]. The spike layers possess the Al composition higher than the quantum barrier so the polarization induced electric field in the spike layers can well reduce the drift velocity of the electrons. The improved capture efficiency is therefore enabled only if the DUV LED is grown along the [0001] orientation. Another effective method to enhance the electron capture rate is to increase the conduction band offset between the quantum barrier and the quantum well, which can be realized by properly increasing the Al composition [10], while the quantum barrier architecture can be further evolved by having the Al composition graded [11]. As has been mentioned previously, the free hole concentration for the Al-rich p-type AlGaN hole injection layer is low which leads to the poor hole injection capability into the MQW region. The poor hole injection is also regarded as the cause of the electron leakage [12]. A promising method for increasing the hole thermionic emission across the p-type electron blocking layer (p-EBL) is to energize the holes by adopting the electric field reservoir [13]. The hole transport can also be favored if the p-AlGaN-based hole injection layer with the stair-cased Al composition is utilized for DUV LEDs [14]. The stair-cased Al composition can be further replaced by the graded Al composition for the AlGaN layer to enhance the hole concentration [15–17]. Besides engineering the hole injection layer, alternative p-EBLs have also been suggested to reduce the hole blocking effect, e.g., inserting a thin AlGaN layer with a lower Al composition [18]. A very important structure for the p-EBL candidate is the superlattice p-EBL. Tremendous research efforts have been made to explore the impact of the GaN/AlGaN superlattice for GaN-based blue LEDs [19–21]. Nevertheless, the AlGaN p-EBL for blue LEDs has the AlN composition lower than 20%, making the hole blocking effect for blue LEDs not as severe as that for DUV LEDs. Therefore, the EQE improvement is smaller than 20% and efficiency droop is still obvious even if the GaN/AlGaN superlattice p-EBL is adopted for blue LEDs. DUV LEDs employ Al-rich p-EBLs, giving rise to an even more challenging hole injection issue [1]. To solve the Al-rich p-EBL-caused hole blocking effect, superlattice p-EBL is also suggested for DUV LEDs, e.g., AlInGaN/AlGaN superlattice p-EBL [22] and AlGaN/AlGaN superlattice p-EBL [23]. However, the experimental proof of the superlattice p-EBL that helps to obtain high and nearly efficiency-droop-free EQE lacks for DUV LEDs at this stage. Therefore, this work experimentally demonstrates the effectiveness of a specifically designed AlGaN/AlGaN superlattice p-EBL in enhancing the EQE and significantly suppressing the efficiency droop for DUV LEDs. The enhanced EQE is well attributed to the improved hole injection into the MQW region while the reduced electron leakage level helps to remarkably suppress the efficiency droop. Detailed mechanism will be presented in this work subsequently.

## Methods/Experimental

**Fig. 1:**
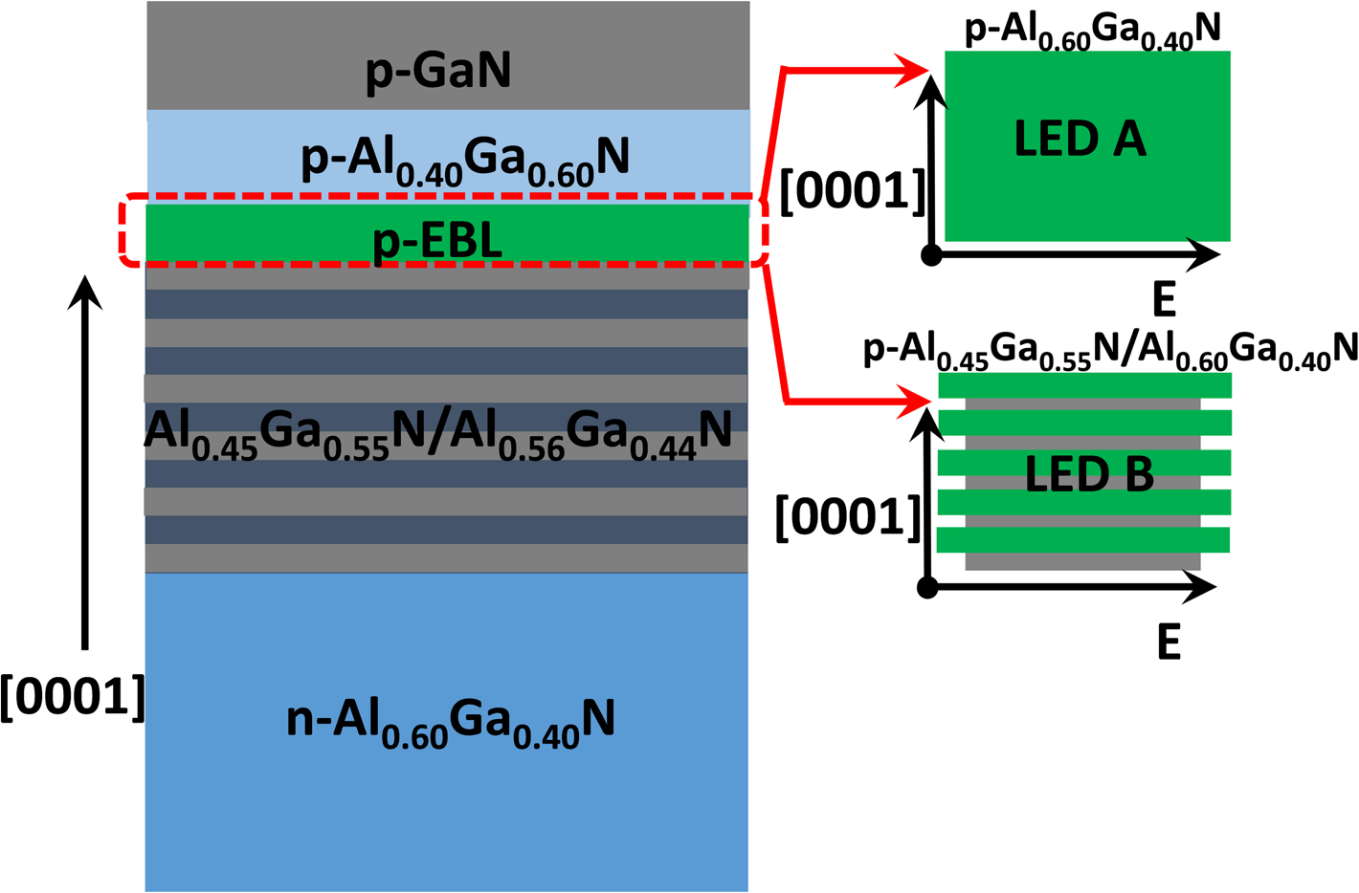
Schematic architectural structures for the studied LEDs. The sketched energy band diagrams for the two p-EBLs are also provided: LED A has the p-Al_0.60_Ga_0.40_N-based EBL and LED B has the p-Al_0.45_Ga_0.55_N/Al_0.60_Ga_0.40_N superlattice EBL. The p-Al_0.45_Ga_0.55_N/Al_0.60_Ga_0.40_N superlattice EBL is specifically designed such that it initiates the thin p-Al_0.45_Ga_0.55_N layer so that the interface for the p-Al_0.45_Ga_0.55_N/Al_0.56_Ga_0.44_N last quantum barrier possesses negative polarization interface charges. *E* means energy level.

The two DUV LED architectures (LEDs A and B as shown Fig. 1) in this work are grown on the AlN template by a metal-organic chemical vapor deposition (MOCVD) system. The 4-μm-thick AlN template is grown on the [0001]-oriented sapphire substrate by using the Hydride Vapor Phase Epitaxy (HVPE) method. We grow 20-period AlN/Al_0.50_Ga_0.50_N superlattice on the AlN template, which serves as the strain-relief layer for the subsequently grown epi-layer. A 2-μm-thick n-Al_0.60_Ga_0.40_N layer that has an electron concentration of 1 x 10^18^ cm^−3^ is grown to provide electrons. The DUV photons are generated by five-period Al_0.45_Ga_0.55_N/Al_0.56_Ga_0.44_N MQWs which have 3-nm-thick Al_0.45_Ga_0.55_N quantum wells and 12-nm-thick Al_0.56_Ga_0.44_N quantum barriers. The MQWs are then capped by a 10-nm-thick AlGaN-based p-EBL. In our experiment, we design and grow two types of p-EBLs for LEDs A and B, respectively. LED A possesses an Al_0.60_Ga_0.40_N-based p-EBL and LED B has a five-period 1-nm Al_0.45_Ga_0.55_N/1-nm Al_0.60_Ga_0.40_N-based p-EBL. Note, our superlattice p-EBL loop starts from the Al_0.45_Ga_0.55_N thin layer after growing the last Al_0.56_Ga_0.44_N quantum barrier. By doing so, the interface of the last quantum barrier/superlattice p-EBL is polarized by yielding negative polarization-induced sheet charges, which helps to deplete the electron accumulation in the last quantum barrier and further suppresses the electron leakage. The p-EBL is then followed by a 50 nm p-Al_0.40_Ga_0.60_N/50 nm p-GaN hole supplier. Lastly, the p-GaN layer is coated with a 10-nm-thick heavily Mg-doped p^+^-GaN layer. The DUV LED wafers are thermally in situ annealed at the temperature of 800 °C in the N_2_ ambient for 15 min to split the H–Mg bonds. The hole concentration is then roughly estimated to be 1 × 10^17^ cm^−3^ and 3 × 10^17^ cm^−3^ for the Al-rich p-AlGaN layer and the p-GaN layer, respectively.

The DUV LED wafers are fabricated into DUV LED chips by following a standard micro-fabrication process. The mesa is obtained by conducting inductively coupled plasma (ICP) etching and the mesa size is 650 × 320 μm^2^. A Ti/Al metal stack is deposited on the n-Al_0.60_Ga_0.40_N layer, which is then annealed in N_2_ for 1 min at the temperature of 900 °C. A Ni/Au current spreading is coated on the mesa surface and then annealed in O_2_ for 5 min at the temperature of 550 °C. Lastly, we deposit Ti/Al/Ni/Au metals simultaneously on the Ti/Al alloy and the Ni/Au current spreading layer serving as the n-electrode and the reflective p-electrode, respectively. The DUV LED chips are flip-chip devices, and the DUV photons are collected from the sapphire side by an integrating sphere.

To better reveal the in-depth origin for the enhanced EQE and the suppressed efficiency droop, numerical calculations are performed by using APSYS package [13, 18]. Important physical parameters that are used to calculate the carrier recombination events and the carrier loss include Shockley-Read-Hall (SRH) recombination lifetime, Auger recombination coefficient, the energy band offset ratio for AlGaN/AlGaN interfaces, and the polarization level for [0001]-oriented III-nitride structures, which are set to 10 ns, 1 × 10^−30^ cm^6^ s^−1^, 50:50, and 40%, respectively [13, 18]. The LEE is set to 10% for bare DUV LED chips with 50-nm-thick absorptive p-GaN layer [5].

## Results and Discussions

**Fig. 2:**
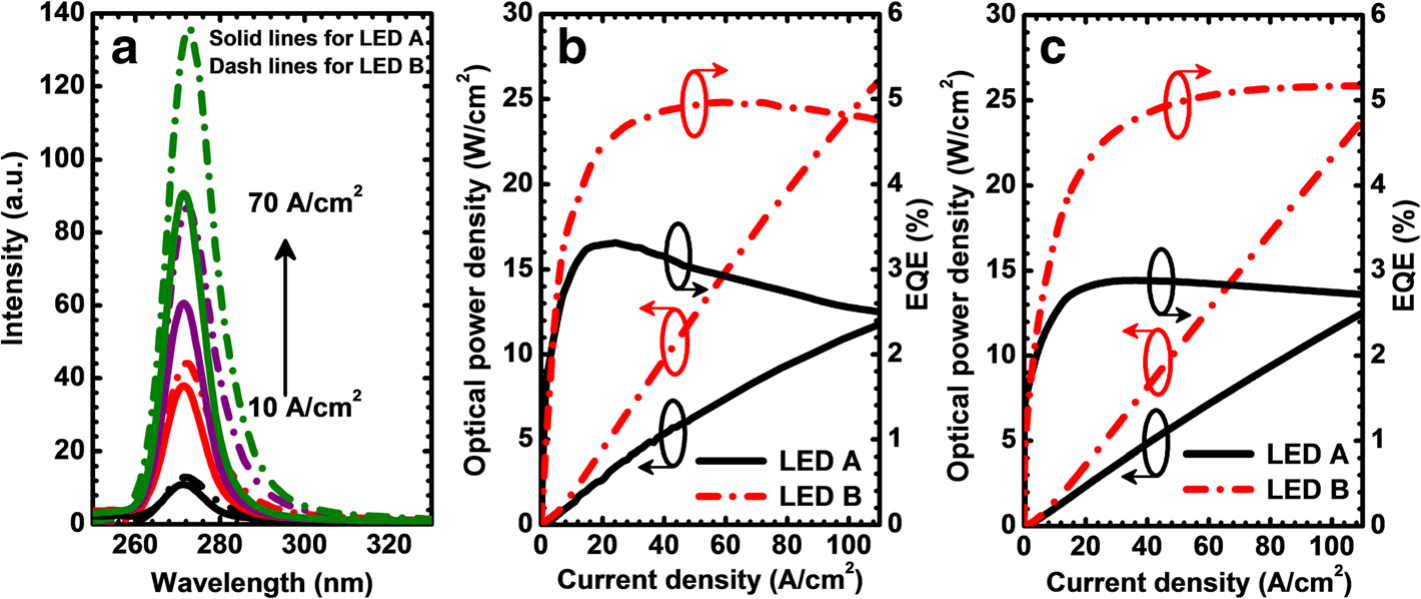
**a** Measured EL spectra at the current density of 10, 30, 50, and 70 A/cm^2^. **b** Measured optical power and EQE. **c** Calculated optical power and EQE for LEDs A and B, respectively

The experimentally measured electroluminescence (EL) spectra at a different current density level for LEDs A and B are presented in Fig. 2a. The EL spectra are collected in the pulsed condition with the duty cycle of 0.1% to avoid the self-heating effect. Figure 2a shows that the peak emission wavelength for both DUV LED devices is ~ 270 nm. The peak emission wavelength is stable within the tested current range because of the elimination of the self-heating effect. The EL intensity for LED B is stronger than that for LED A. Figure 2b demonstrates the optical power and the EQE as the function of the injection current density, which illustrates that the EQE is enhanced by ~ 90%. Furthermore, the efficiency droop levels are ~ 24 and ~ 4% for LEDs A and B at the current density level of 110 A/cm^2^, respectively [droop = (*EQE*_max_ − *EQE*_*J*_)/*EQE*_max_, in which *EQE*_max_ and *EQE*_*J*_ denote the maximum EQE and the EQE at the current density of *J*]. Figure 2c presents the numerically calculated optical power density and the EQE in terms of the injection current density. The numerically calculated results and the experimentally measured ones agree well with each other, such that LED B shows the enhanced EQE and a substantially reduced efficiency droop level. The agreement between Fig. 2b and 2c well validates the physical models and the parameters we set for computations.

**Fig. 3:**
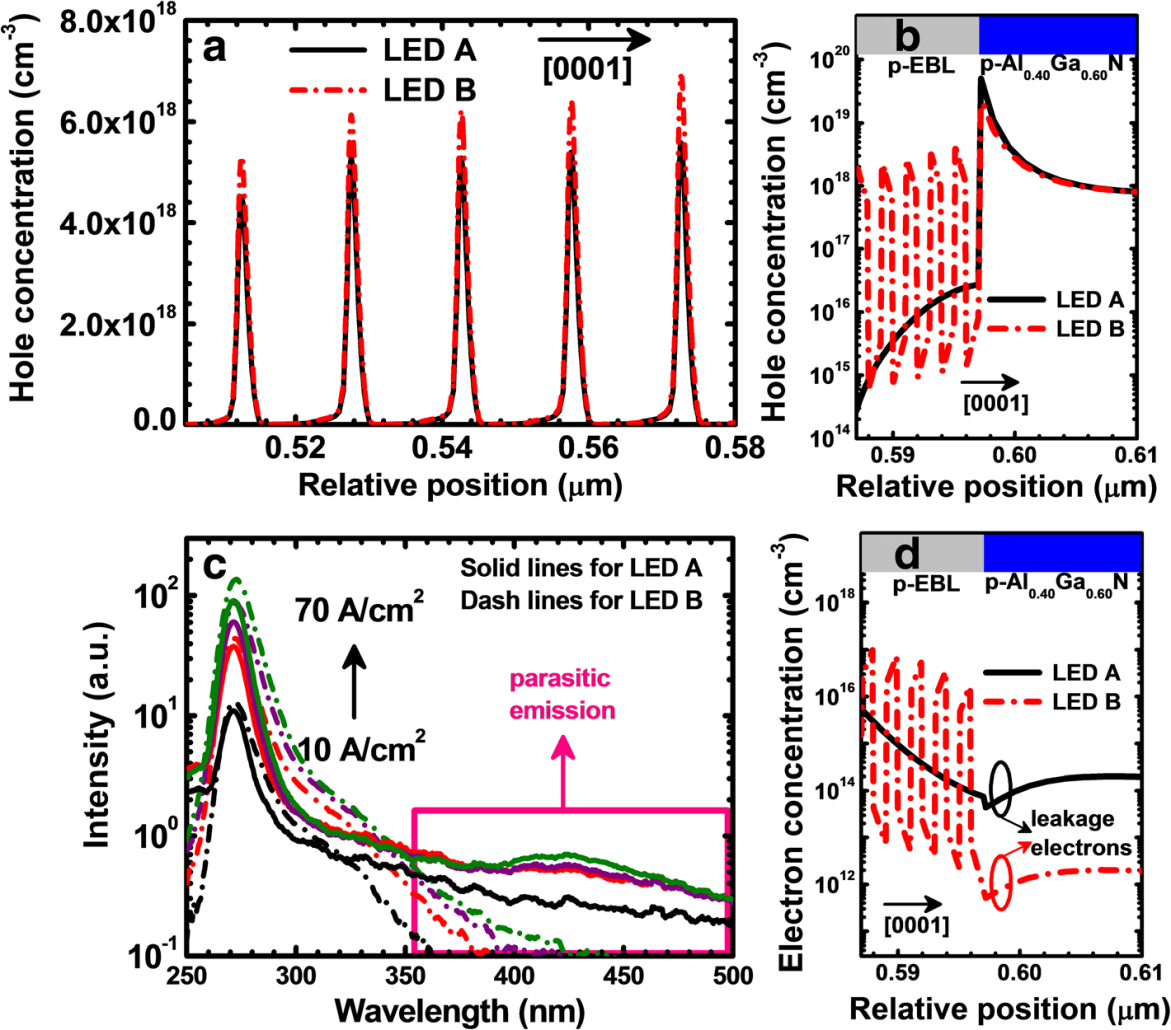
Numerically calculated hole concentration profiles **a** in the MQWs and **b** in the p-type hole injection layers for LEDs A and B, respectively; **c** experimentally measured EL spectra in semi-log scale at the current density of 10, 30, 50, and 70 A/cm^2^ for LEDs A and B, respectively; **d** numerically calculated electron concentration levels in the p-type hole injection layers for LEDs A and B. Numerically calculated data are collected at the current density of 50 A/cm^2^

The two DUV LEDs differ from each other only in the p-EBL. Therefore, it is required to investigate the role of the superlattice p-EBL in improving the optical performance for LED B. Figure 3a presents the hole concentration profiles across the MQW region for LEDs A and B at the current density of 50 A/cm^2^. It is shown that the hole concentration level within the MQWs for LED B is higher than that for LED A. As has been reported, the p-EBL reduces the electron leakage level while simultaneously hindering the hole injection [24]. A useful approach to reduce the hole blocking effect is to increase the hole concentration within the p-EBL region, which then helps to decrease the valence band barrier height [25]. Figure 3b then shows the hole concentration levels in the p-EBLs and the p-Al_0.40_Ga_0.60_N layers for LEDs A and B at the current density of 50 A/cm^2^. The average hole concentration in the superlattice p-EBL for LED B is much higher than that for LED A by two orders of magnitude. The larger hole concentration in the superlattice p-EBL is well attributed to the excellent hole transport. Interestingly, if we further look into Fig. 3b, we find that the hole concentration at the p-EBL/p-Al_0.40_Ga_0.60_N interface become lower for LED A, which also reflects the smoother hole injection efficiency through the superlattice p-EBL for LED B.

As has been mentioned previously, the efficiency for LEDs is closely associated with the electron leakage level. Therefore, we show the measured EL spectra for LEDs A and B in a semi-log scale (see Fig. 3c) to indicate the detailed information regarding the parasitic luminescence. The peak emission wavelength for the parasitic luminescence is centered at ~ 425 nm, which may arise from the deep levels associated with Mg dopants [26]. The intensity of the parasitic luminescence for LED B is stronger than that for LED A, and it is speculated that more carriers recombine at the deep levels. In our experiment, the p-type hole injection layers for both DUV LED architectures are not engineered, and the hole concentration level in the hole injection layers shall be similar. Therefore, it is identified that electrons that escape from the MQW region possess a higher concentration in the hole injection layer for LED B than those for LED A. Our suggestions are further supported by Fig. 3d that shows the electron concentration profiles in the p-type hole injection layers for LEDs A and B at the current density of 50 A/cm^2^. This also means that the electron leakage current has been significantly reduced thanks to the superlattice p-EBL for LED B.

**Fig. 4:**
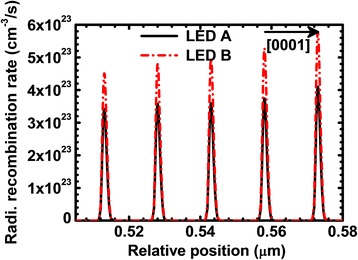
Numerically computed profiles of the radiative recombination rate for LEDs A and B. Data are collected at the current density level of 50 A/cm^2^

We then present the computed profiles of the radiative recombination rate for LEDs A and B in Fig. 4 which are collected at the current density level of 50 A/cm^2^. It is inferred that radiative recombination rate for LED B is stronger than that for LED A thanks to the proposed superlattice p-EBL, which even more favors the hole injection into the MQW region and suppresses the electron leakage level in the meantime.

**Fig. 5:**
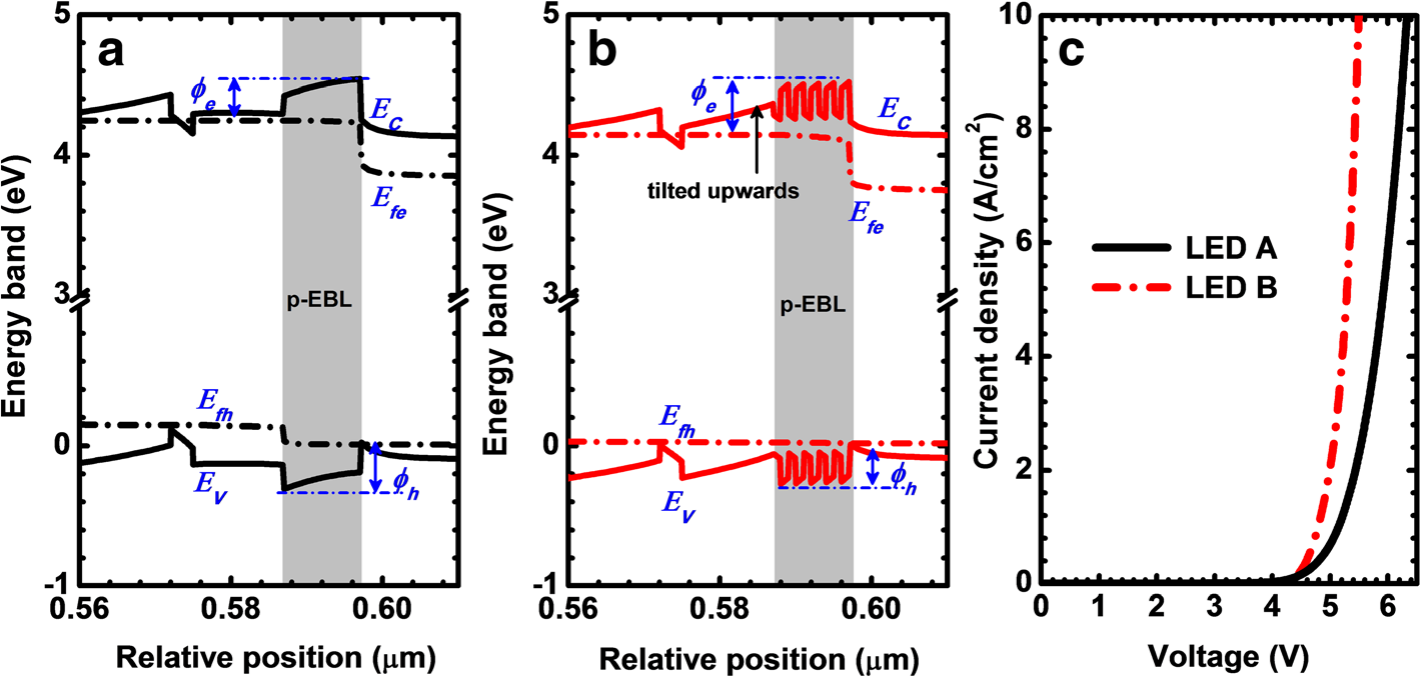
Numerically computed energy band profiles in the vicinity of **a** bulk AlGaN-based p-EBL for LED A, **b** superlattice p-EBL for LED B, and **c** measured current density in terms of the applied bias for LEDs A and B. Data for **a** and **b** are calculated at the current density of 50 A/cm^2^. *E*_*C*_, E_V_, *Ø*_*e*_, and *Ø*_*h*_ denote the conduction band, the valence band, and the effective barrier heights for conduction band and valence band, respectively

Figure 5a and 5b show the energy band in the vicinity of the p-EBLs for both DUV LED devices. The energy bands are calculated at the current density of 50 A/cm^2^. As has been reported by Zhang et al. [27], the strong polarization induced positive charges at the last quantum barrier/p-EBL interface can significantly attract electrons, giving rise to the high local electron concentration. The high local electron concentration can reduce the effective conduction band barrier height (*Ø*_*e*_) for the p-EBL which is ~ 295 meV for LED A. If the bulk AlGaN based p-EBL is replaced by the specific superlattice p-EBL in this work (i.e., the superlattice p-EBL loop starts from the thin AlGaN layer with a smaller energy band gap than the last AlGaN quantum barrier), the conduction band for the last quantum barrier is titled upwards (see Fig. 5b), and this favors an electron depletion in the last quantum barrier which then increases the *Ø*_*e*_ to ~ 391 meV and enables a smaller electron escape by means of thermionic emission [28]. Furthermore, the superlattice p-EBL facilitates the intra-band tunneling process for holes, as the result of which the hole concentration in the p-EBL also becomes higher (see Fig. 3b) The enhanced hole concentration in the p-EBL for LEDs tends to reduce the effective valence band barrier height (*Ø*_*h*_) [25], i.e., the values of *Ø*_*h*_ are ~ 324 meV and ~ 281 meV for LEDs A and B, respectively at the current density of 50 A/cm^2^. The even smaller *Ø*_*h*_ for LED B in turn favors the thermionic emission for holes. It is worth noting that the superlattice p-EBL may also cause the intra-band tunneling for electrons. Fortunately, the improved hole concentration in the MQW can better consume electrons by radiative recombination, which also contributes to alleviate the electron leakage [12]. Because of the more favored hole injection and the even stronger recombination current that is produced by the radiative recombination process taking place in the MQW region, the forward voltage becomes smaller for LED B than that for LED A according to Fig. 5c.

## Conclusions

To summarize, this work has reported a specific superlattice p-EBL for DUV LEDs, which can maintain both the promoted hole injection efficiency and the decreased electron leakage into the passive p-type hole injection layer. Therefore, both numerically and experimentally, the improved EQE and the remarkably suppressed efficiency droop are obtained. We strongly believe that the proposed DUV LED structure is very promising for realizing high-efficiency DUV LEDs and the device physics revealed by this work introduces more understanding to the III-nitride-based optoelectronic community.

## Abbreviations

APSYS: Advanced Physical Models of Semiconductor Devices
DUV: Deep ultraviolet light-emitting diodes
EL: Electroluminescence
EQE: External quantum efficiency
HVPE: Hydride Vapor Phase Epitaxy
ICP: Inductively Coupled Plasma
IQE: Internal quantum efficiency
LEE: Light extraction efficiency
MOCVD: Metal-organic chemical vapor deposition
MQWs: Multiple quantum wells
p-EBL: p-type electron blocking layer
TDD: threading dislocation density
